# Atypical Fibroxanthoma-Like Melanoma: A Rare Subtype of High-Cumulative Sun Damage Melanoma With Partial Dedifferentiation and an Aggressive Molecular Profile

**DOI:** 10.7759/cureus.79414

**Published:** 2025-02-21

**Authors:** Igor Shendrik, Svetlana Bobkova, Eli P Oldham, Jennifer Roberts

**Affiliations:** 1 Dermatopathology Section, Regional Medical Laboratory, Inc. & Pathology Laboratory Associates, Inc., Tulsa, USA; 2 School of Biomedical Sciences, Oklahoma State University Center for Health Sciences, Tulsa, USA; 3 Office of Medical Student Research, Oklahoma State University Center for Health Sciences, Tulsa, USA; 4 Dermatology, Jennifer Roberts Dermatology, Norman, USA

**Keywords:** afx-like melanoma, atm mutation, dedifferentiated melanoma, fat1 mutation, pbrm1 mutation

## Abstract

We report a rare case of atypical fibroxanthoma (AFX)-like melanoma on the scalp of a 75-year-old man. A biopsy of an erythematous, tender nodule on the left posterior parietal scalp revealed an ulcerated nodular lesion composed of epithelioid and spindle cells with pleomorphic nuclei arranged in a fascicular pattern. The tumor's architectural and cytologic features, including the presence of an epidermal collarette, marked anisonucleosis, and numerous atypical mitoses, closely resembled those of AFX. Immunohistochemical analysis demonstrated SOX10 positivity and negativity for other melanocytic markers. Molecular profiling confirmed the diagnosis of melanoma and identified mutations in the TERT promoter, NRAS, NF1, PBRM1, FAT1, and ATM genes. The tumor was categorized as Class 2B by the DecisionDx-Melanoma test (Castle Biosciences, Friendswood, Texas), indicating a high risk of recurrence and metastasis. Sentinel lymph node excision revealed metastatic melanoma in two of the five examined nodes, further supporting the aggressive biological nature of this neoplasm. This case illustrates the phenomenon of melanoma dedifferentiation, resulting in an AFX-like appearance. We discuss potential molecular mechanisms underlying this transformation, with mutations in NF1, PBRM1, and FAT1 likely contributing to the tumor's atypical morphology and loss of melanocytic markers. The high tumor mutational burden and aggressive molecular profile align with the reported poor prognosis of dedifferentiated melanomas. Recognizing this rare variant is critical for accurate diagnosis, effective patient management, and prognosis assessment.

## Introduction

Atypical fibroxanthoma-like melanoma (AFX-like melanoma) exhibits histologic and immunophenotypic features resembling atypical fibroxanthoma (AFX), a pleomorphic dermal neoplasm commonly seen in elderly patients with sun-damaged skin [1-3]. The scarcity of documented cases in the literature underscores the complexity of this melanoma subtype. Clinically, AFX-like melanomas typically present as nodular, amelanotic lesions that lack distinctive features suggestive of melanocytic origin. Histopathologically, these melanomas exhibit pleomorphic, spindle-shaped, or multinucleated giant cells arranged in storiform or haphazard patterns. A key diagnostic challenge arises from the complete or partial loss of expression of conventional melanocytic markers, including S100 protein, SRY-Box transcription factor 10 (SOX10), melanoma antigen A (Melan-A), and human melanoma black-45 (HMB-45).

Genetically, these tumors harbor mutations characteristic of melanoma, particularly those associated with chronic sun damage. These mutations include alterations in neuroblastoma RAS (NRAS), telomerase reverse transcriptase (TERT), and neurofibromatosis type 1 (NF1) [4,5]. Additionally, a high tumor mutational burden (TMB), which is common in these tumors, reflects sun-damaged skin and likely contributes to their aggressive behavior. Furthermore, these tumors may present as biphenotypic neoplasms, raising the differential diagnosis of a collision tumor. However, advanced molecular profiling has revealed shared mutational landscapes between conventional melanoma components and dedifferentiated AFX-like areas, providing evidence that these lesions represent a biphenotypic presentation rather than a true collision tumor. While some authors suggest that the presence of de-differentiation or trans-differentiation does not necessarily result in a profoundly worse outcome for patients with these tumors, the limited number of reported cases precludes definitive prognostication [5]. This report seeks to expand the limited literature by presenting a newly documented case of AFX-like melanoma. It correlates the clinical presentation with histopathologic, immunohistochemical, and genetic findings and discusses the implications for diagnosis and management.

## Case presentation

**Figure 1:**
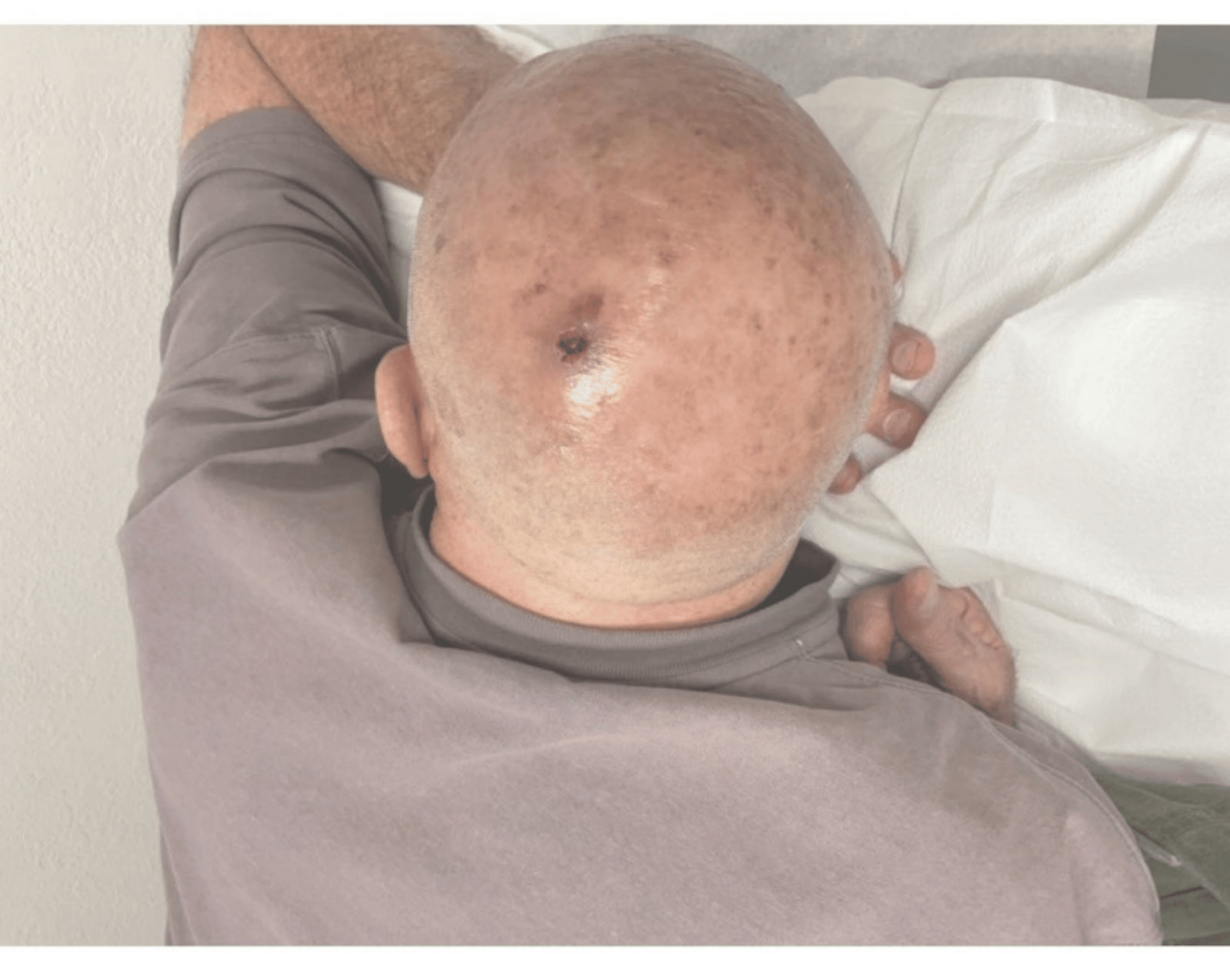
Patient image at the time of admission Single erythematous nodule with central ulceration located on the left posterior parietal scalp. The surrounding scalp shows signs of sun damage, including multiple lentigines and an atrophic, shiny appearance.

A 75-year-old man was seen in a dermatology office, presenting with an erythematous tender nodule with a hyperkeratotic scale on his left posterior parietal scalp (Figure 1). The 1-cm lesion had persisted for about a year. The initial clinical differential diagnosis encompassed squamous cell carcinoma versus irritated seborrheic keratosis.

**Figure 2:**
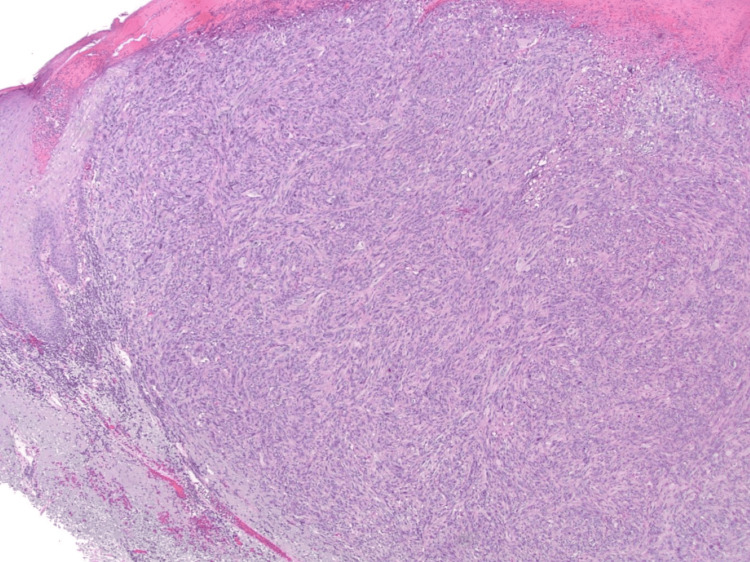
Low-power histopathological image (X20) The image shows sun-damaged skin with an ulcerated spindle cell tumor and surrounding epidermal collarette.

**Figure 3:**
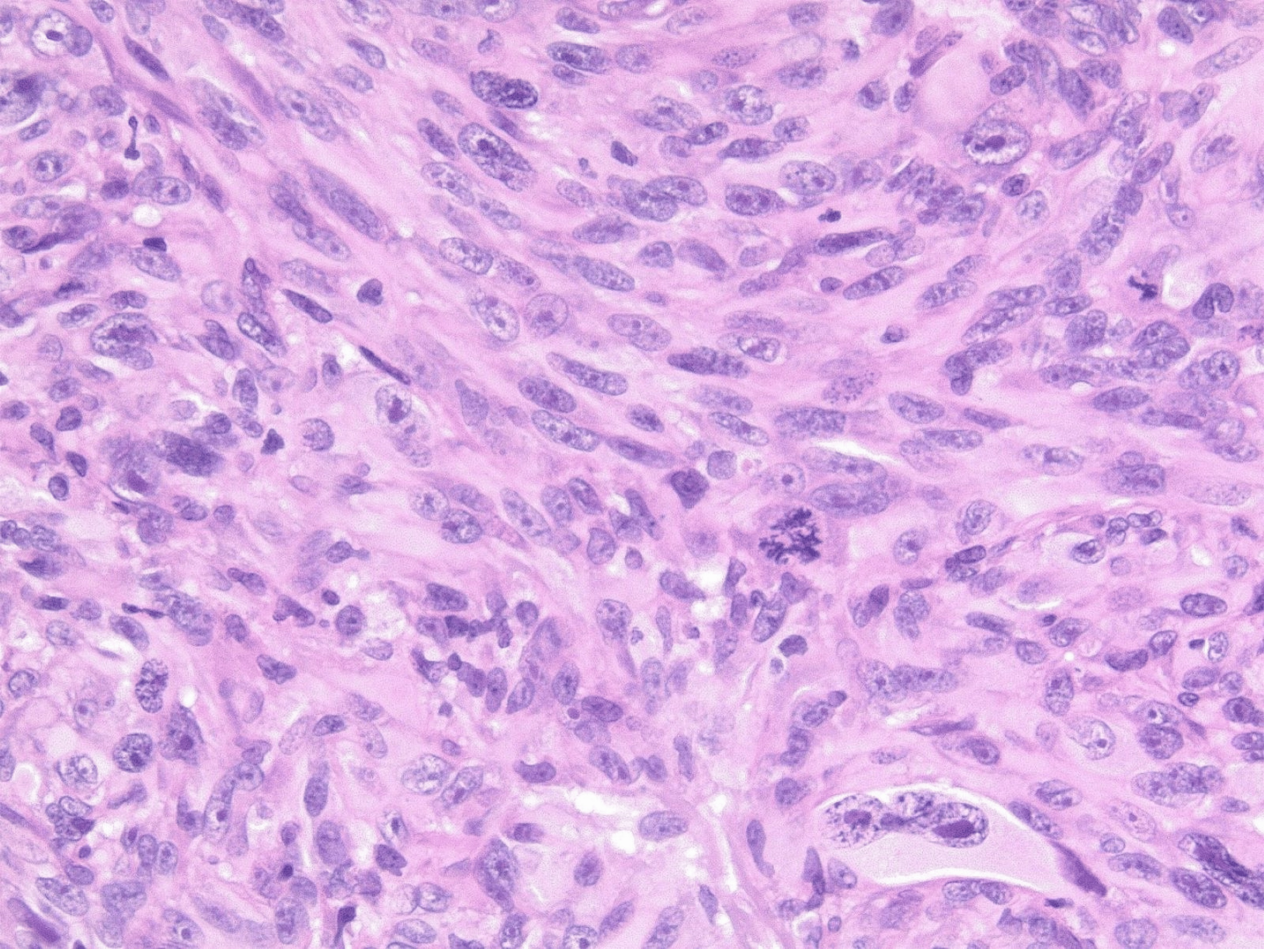
High-power histopathological image (X200) The image shows a spindle cell tumor with marked anisonucleosis, multinucleation, and atypical mitotic figures.

**Figure 4:**
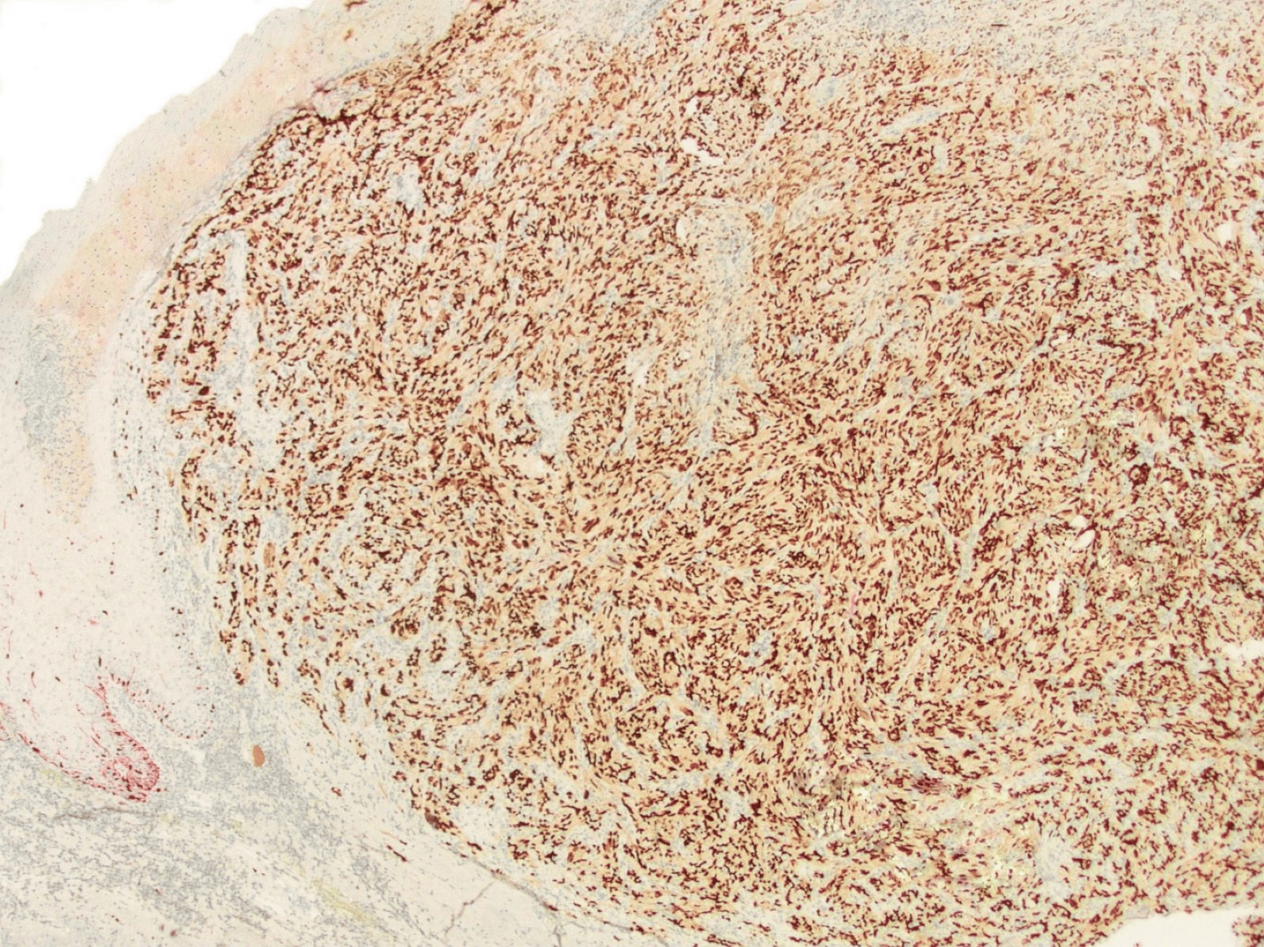
Histopathological image obtained with double immunohistochemical staining (X20) Double immunohistochemical staining with Melan-A (red cytoplasmic stain) and SOX10 (dark brown nuclear stain), demonstrated scattered, low-density positive junctional melanocytes at the tumor periphery. The invasive component shows SOX10 positivity, with a loss of Melan-A expression.

Histopathological examination of hematoxylin and eosin-stained sections revealed an ulcerated nodular lesion surrounded by an epidermal collarette (Figure 2). The tumor comprises epithelioid and spindle cells with pleomorphic, hyperchromatic nuclei, arranged in fascicular and haphazard patterns (Figure 3). Giant multinucleated cells are seen. The lesion shows up to 8 mitoses per mm^2^ with frequent atypical division figures (Figure 4). The tumor nodule was surrounded by chronic inflammatory infiltrates but the lesional center did not contain lymphocytic aggregates.

**Figure 5:**
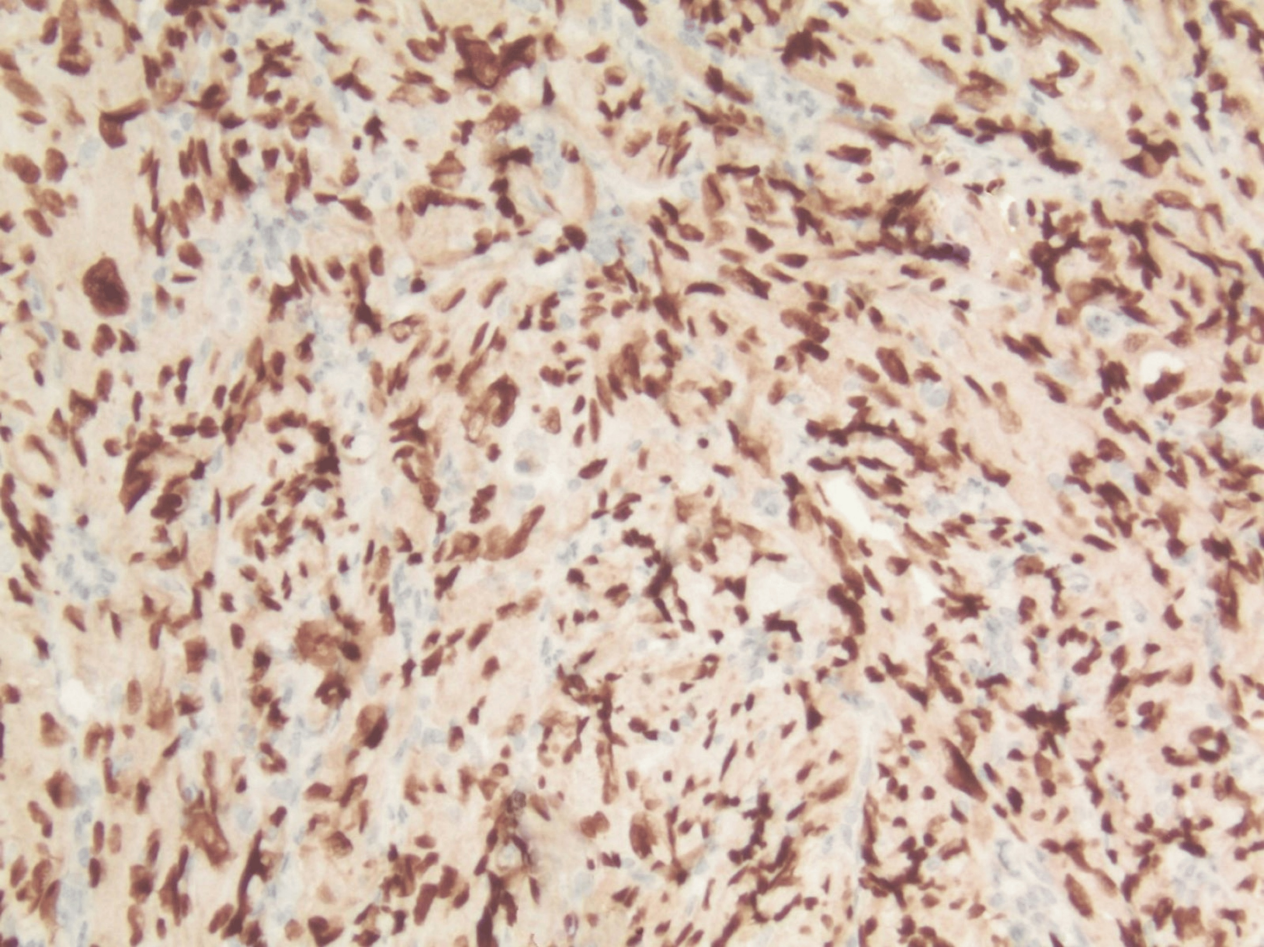
Histopathological image obtained with double immunohistochemical staining (X200) Double immunohistochemical staining with Melan-A (red cytoplasmic stain) and SOX10 (dark brown nuclear stain), demonstrated positivity of the tumor with SOX10 and loss of Melan-A expression.

Immunohistochemical stains demonstrated positivity of the tumor with sex-determining region Y-box 10 (SOX10) and negative staining with smooth muscle actin (SMA), Cluster of Differentiation 10 (CD10), protein 40 (P40), cytokeratin 5/6 (CK5/6), and melanin (Figures 4, 5). The tumor was diagnosed as ulcerated melanoma, not otherwise specified, invasive to a depth of 3 mm (pathologic tumor stage 3b, pT3b). Unusual tumor morphology, reminiscent of atypical fibroxanthoma (AFX), was noted, and the lesion was subsequently sent for genetic testing.

Molecular profiling was performed using the Caris Molecular Intelligence test (Caris Life Sciences, Irving, Texas) [6]. The test confirmed the diagnosis of melanoma with 99% confidence using the Caris Genomic Probability Score-artificial Intelligence (GPSai) [6]. The Caris Molecular Intelligence test results were confirmatory of the melanoma diagnosis. Details of the test results are provided in Table 1. 

**Table 1:** Caris Life Sciences Molecular Intelligence Test – Genetic and Biomarker Findings TERT (Telomerase Reverse Transcriptase), PD-L1 (Programmed Death-Ligand 1), NRAS (Neuroblastoma RAS Viral (v-ras) Oncogene Homolog), NF1 (Neurofibromin 1), MSI (Microsatellite Instability), NTRK (Neurotrophic Tyrosine Receptor Kinase), RET (Rearranged during Transfection), ALK (Anaplastic Lymphoma Kinase), BRAF (B-Raf Proto-Oncogene), CDKN2A (Cyclin-Dependent Kinase Inhibitor 2A), HER2/Neu (Human Epidermal Growth Factor Receptor 2), KIT (V-Kit Hardy-Zuckerman 4 Feline Sarcoma Viral Oncogene Homolog), MAP2K1 (Mitogen-Activated Protein Kinase Kinase 1), MAP2K2 (Mitogen-Activated Protein Kinase Kinase 2), MTAP (Methylthioadenosine Phosphorylase), PTEN (Phosphatase and Tensin Homolog), RAC1 (Rac Family Small GTPase 1), ROS1 (C-ros Oncogene 1), TMB (Tumor Mutational Burden), LOH (Loss of Heterozygosity), PBRM1 (Polybromo 1), FAT1 (Fat Tumor Suppressor 1), ATM (Ataxia-Telangiectasia Mutated), VUS (Variant of Uncertain Significance), BRCA1 (Breast Cancer 1), Her2/Neu (Human Epidermal Growth Factor Receptor 2), ATP6AP2 (ATPase H+ Transporting Accessory Protein 2), CUL3 (Cullin 3), MGA (Max-Interacting Protein), NPM1 (Nucleophosmin 1), PLCB4 (Phospholipase C Beta 4), PRDM6 (PR Domain Containing 6), PRKD1 (Protein Kinase D1), RASA1 (RAS P21 Protein Activator 1), REST (RE1-Silencing Transcription Factor), SMARCA2 (SWI/SNF Related, Matrix Associated, Actin Dependent Regulator of Chromatin Subfamily A Member 2), STAG2 (Stromal Antigen 2), SUZ12 (Suppressor of Zeste 12), COL2A1 (Collagen Type II Alpha 1 Chain), MDH2 (Malate Dehydrogenase 2).

Characteristics	Genetic and Biomarker Findings
Cancer-Type Relevant Biomarkers	TERT Promoter: Pathogenic variant, c.-124C>T
	PD-L1 (SP142): Positive, 2+, 90% (Immunohistochemistry)
	NRAS: Pathogenic variant in exon 2, p.G13D
	NF1: Variant of uncertain significance (Exon 25, p.P1084S)
	Note: No abnormalities were identified in MSI, NTRK1/2/3, RET, ALK, BRAF, CDKN2A, HER2/Neu, KIT, MAP2K1, MAP2K2, MTAP, PTEN, RAC1, or ROS1
Genomic Signatures	Tumor Mutational Burden: High (46 mutations/Mb)
	Microsatellite Instability: Stable
	Genomic Loss of Heterozygosity: Low (4%, threshold >16%
Pathogenic or Likely Pathogenic Alterations	TERT: Pathogenic variant, c.-124C>T, detected in 21% of tumor cells
	NRAS: Pathogenic variant, exon 2 (p.G13D), detected in 53% of tumor cells
	PBRM1: Pathogenic variant, p.R710*, exon 17, c.2128C>T, detected in 25% of tumor cells; Pathogenic variant, p.K1326fs, exon 26, c.3978_3979delinsT, detected in 24% of tumor cells
	FAT1: Pathogenic variant, p.Q301, exon 2, c.901C>T, detected in 26% of tumor cells
	ATM: Likely pathogenic variant, p.R1466*, exon 29, c.4396C>T, detected in 24% of tumor cells
Variants of Uncertain Significance (VUS)	BRCA1: p.D399E, exon 10, c.1191C>A, detected in 29% of tumor cells; c.5153-6C>T, exon 18, c.5153-6C>T detected in 26% of tumor cells
	ATM: c.6095+delA, exon 41, detected in 45% of tumor cells
	NF1: p.P1084S, DNA alteration c.3250C>T, detected in 29% of tumor cells
	NTRK3: p.L364F, DNA alteration c.1090C>T
	Immunohistochemistry: Her2/Neu- negative; PD-L1- positive
Genes with Indeterminate Results by Tumor DNA Sequencing	ATP6AP2, CUL3, MGA, NPM1, PLCB4, PRDM6, PRKD1, RASA1, REST, SMARCA2, STAG2, SUZ12, COL2A1, MDH2

The case was also analyzed using the DecisionDx-Melanoma test (Castle Biosciences, Friendswood, Texas) [7]. The tumor was classified as Class 2B melanoma with the highest possible 31-gene expression profile (31-GEP) score of 1.0 (range: 0.0-1.0). Personalized risk estimates were provided for five-year outcomes: melanoma-specific survival was 76.6%, distant metastasis-free survival was 44.4%, recurrence-free survival was 30.4%, and the likelihood of sentinel lymph node positivity was 32.0%.

A wide local excision of the melanoma was performed 1.5 months after the initial biopsy. The excision was carried out with a 2-cm peripheral margin, extending to the loose areolar fascia at the deep margin, resulting in a final defect with a circumferential diameter of 6 cm. A sentinel lymph node from the left level V nodal basin was identified using a gamma probe and subsequently excised.

Histologic examination of the excised tissue revealed residual invasive melanoma and melanoma in situ, both of which were excised with adequate margins. A gross examination of the sentinel lymph node specimen identified five lymph nodes. Two of the five lymph nodes demonstrated a few atypical human melanoma black 45 (HMB-45)-positive melanocytes. Some of these melanocytes were localized subcapsularly, while others were scattered. The melanocytes displayed a cytologic appearance identical to that of the invasive melanoma, confirming metastatic disease in two of the five lymph nodes (2/5).

## Discussion

Based on the 2018 World Health Organization (WHO) classification of cutaneous melanocytic neoplasms, melanomas arising on sun-exposed skin are categorized into two main pathways: low cumulative sun damage (low-CSD) and high cumulative sun damage (high-CSD) melanomas. Low-CSD melanomas often harbor BRAF V600E mutations and have a lower TMB. In contrast, high-CSD melanomas frequently lack BRAF V600E mutations but may have alterations in genes such as NRAS, NF1, and KIT while exhibiting a higher TMB. High-CSD melanomas are characterized by a high number of point mutations, predominantly consisting of the "ultraviolet (UV) signature" (cytosine to thymine transitions at dipyrimidine sites) [4].

The degree of cumulative sun damage correlates with the TMB, with high-CSD melanomas averaging 30 mutations per megabase (Mb) [4]. Desmoplastic melanoma, a subtype of high-CSD melanoma, demonstrates an even higher TMB, averaging 62 mutations per Mb [4]. The Cancer Genome Atlas (TCGA) classification categorizes melanomas into four main subtypes based on their genetic profiles, particularly their driver mutations [8]. High-CSD melanomas often demonstrate RAS-mutated or NF1-mutated profiles in this classification system [8]. NF1 mutations are also more frequent in desmoplastic melanomas [4].

Recent advances in genetic testing have brought attention to melanoma dedifferentiation and transdifferentiation [5]. These processes are significant both due to their complexity and potential for misdiagnosis and because of the practical ability to establish tumor lineage in divergent populations of neoplastic cells. Melanoma dedifferentiation refers to the tumor's progression toward a less differentiated state, often including the loss of typical melanocytic markers such as S100 protein, SOX10, Melan-A, and HMB-45. In some cases, melanoma undergoes further or abrupt transdifferentiation, acquiring a phenotype that is morphologically and immunohistochemically identical to other tumor types [5].

Some publications describe this process as sarcomatoid dedifferentiation [9] or sarcomatoid undifferentiated melanoma [10]. While the phenomenon of divergent differentiation in melanomas is well documented, diagnosing individual cases can be challenging [11]. This has prompted recent studies on melanoma presentations that mimic atypical fibroxanthoma (AFX) [1-3]. These cases are characterized by histologic tumor appearance, complete or partial loss of melanoma markers, and, in some instances, acquisition of the typical AFX immunohistochemical staining pattern. Defined as AFX-like melanoma, this rare subtype poses significant diagnostic challenges [5].

The phenomenon of melanomas mimicking AFX is documented in several reports. Sangüeza and Zelger (2007) described AFX-like melanomas resembling pleomorphic storiform patterns typical of AFX but retaining certain melanocytic molecular characteristics [1]. The authors emphasized their poorer prognosis compared to AFX, underlining the need for precise diagnosis [5]. Similarly, Wang and Guo (2021) and others highlighted the diagnostic challenges of these rare melanoma subtypes, which can present as amelanotic or multinucleated lesions, further complicating identification [2].

Genetic analyses have shown that most somatic mutations in AFX-like melanomas overlap with conventional melanoma components, arguing against the collision tumor hypothesis and supporting a clonal relationship between these elements [5]. For example, Ferreira et al. (2021) demonstrated shared mutations across primary, dedifferentiated, and metastatic components of such tumors [5].

The case presented here exemplifies the diagnostic challenges posed by dedifferentiated melanomas, particularly those with AFX-like features. This tumor's genetic profile and morphological characteristics align with the high-CSD melanoma subtype as defined by the 2018 WHO Classification [4]. Our patient's melanoma, which arose on the sun-exposed skin of the scalp in an elderly individual, demonstrates key features of high-CSD melanomas. These include a high tumor mutational burden (46 mutations per Mb), the presence of NRAS and NF1 mutations, and the absence of BRAF V600E mutations. This genetic profile is consistent with the RAS-mutated or NF1-mutated subtypes described in the TCGA classification [8]. The tumor's AFX-like morphology and partial loss of melanocytic markers (Melan-A negativity with retained SOX10 expression) represent a phenomenon of melanoma dedifferentiation.

AFX-like melanomas are characterized by mutations in the TERT promoter, NRAS, NF1, and TP53, with high TMB frequently observed​​ [5]. Additional findings include chromosomal aberrations such as loss of chromosome 9p, loss of chromosome 10q, and gain of chromosome 8q​​ [5]. Given the above literature review, it is prudent to consider any areas resembling AFX adjacent to melanoma as potentially representing a transdifferentiated melanoma until proven otherwise by genetic testing, regardless of the immunohistochemical profile.

In this case, we encountered melanoma with clinical features (scalp of an older male), architectural features (ulcerated nodular lesion with epidermal collarette), and cytologic features (large, atypical cells with numerous atypical mitoses) resembling AFX. The lesion lacked precursor melanoma and showed a loss of Melan-A expression, with preservation of SOX10 expression. These clinical, morphologic, and immunohistochemical features fit well with the partial dedifferentiation of melanoma, resulting in an AFX-like appearance.

Molecular profiling was performed using the Caris Molecular Intelligence test (Caris Life Sciences, Irving, Texas) [6]. The test confirmed the diagnosis of melanoma with 99% confidence using the Caris Genomic Probability Score-artificial Intelligence (GPSai). Further evaluation of the tumor by Caris Molecular Intelligence revealed several mutations, including the NF1 variant of uncertain significance (VUS), NRAS, PBRM1, FAT1, TERT Promoter, and ATM. 

Typically, NF1 and NRAS mutations are considered mutually exclusive in melanomas, as both affect the RAS/MAPK pathway [5]. The NRAS (p.G13D) mutation identified in this case is a known activating mutation that can drive melanoma progression through the RAS/MAPK pathway [5]. Given this context and the presence of a strong driver mutation in NRAS, we hypothesize that the NF1 p.P1084S variant is likely a passenger mutation rather than a driver in this case. This interpretation is further supported by the Caris Molecular Intelligence classification of the NF1 VUS.

The PBRM1 truncating mutations (p.R10* and p.K1326fs) and FAT1 mutation (p.Q301) are particularly intriguing in the context of the AFX-like morphology. PBRM1, a component of the SWItch/Sucrose Non-Fermentable (SWI/SNF) chromatin remodeling complex, plays a crucial role in regulating gene expression and cellular differentiation [12]. The loss of PBRM1 function could lead to dysregulation of genes involved in maintaining melanocytic identity. Concurrently, the FAT1 mutation might alter cell adhesion properties, [13] potentially contributing to the mesenchymal-like appearance characteristic of AFX. The combination of these mutations could explain the loss of typical melanocytic features and the adoption of an AFX-like phenotype, aligning with previous literature suggesting these genes contribute to dedifferentiation and atypical morphologic changes in tumors [12,13].

The TERT promoter mutation, while not specific to AFX-like morphology, is a frequent driver mutation in melanoma [4]. It supports telomerase reactivation and cellular immortality, common features in aggressive melanomas. This mutation contributes to the overall aggressive nature of the tumor, even if it doesn't directly explain the AFX-like appearance [4]. The ATM mutation (p.R1466) is another significant finding in this case. ATM plays a crucial role in DNA damage response and repair, and its mutation can lead to increased genomic instability and high mutational burden [14].

The current case was also shown to have a high TMB of 46 mutations/Mb, placing it right between the typical TMB of high-CSD melanoma and desmoplastic melanoma [4]. It is of interest that typical genetic features of AFX include mutations in TP53, TERT promoter, CDKN2A, and NOTCH1, along with a high TMB, some of which show overlap with the genetic profile of this melanoma, likely due to common site of origin on sun-damaged skin [15].

In summary, the genetic profile of this AFX-like melanoma case presents a complex interplay of mutations affecting various cellular processes. The NRAS mutation likely serves as the primary driver, with the NF1 VUS being a probable passenger mutation. The PBRM1 and FAT1 mutations offer a plausible explanation for the AFX-like morphology through their effects on cellular differentiation and adhesion [12,13]. The TERT promoter and ATM mutations further contribute to the aggressive cytologic features of the tumor [4,14].

This case was classified as Class 2B by Castle Biosciences' DecisionDx-Melanoma test, indicating a high-risk tumor biology with increased risk for recurrence and metastasis. In Castle Biosciences' framework, a Class 2B classification corresponds to a significantly elevated hazard ratio for both melanoma-specific mortality and recurrence, underscoring the aggressive potential of this tumor [7]. The subsequent excision of the tumor with lymph node sampling demonstrated metastatic disease in two of the five examined sentinel lymph nodes, supporting the results of genetic prognostication and highlighting the aggressive nature of this neoplasm.

## Conclusions

We describe a rare case of AFX-like melanoma with partial dedifferentiation and AFX-like histologic appearance. The tumor lineage and aggressive potential was confirmed by extensive genetic testing revealing a high tumor mutational burden and pathogenic mutations in TERT, NRAS, and NF1, alongside alterations in PBRM1 and FAT1, which may contribute to the tumor's unique morphology. Classified as Class 2B by the DecisionDx-Melanoma test and demonstrating sentinel lymph nodes metastases, this tumor carries a high risk for poor outcomes. The morphologic and genetic findings support melanoma's capacity for dedifferentiation toward AFX-like lesions. Future studies should explore the role of these mutations in pathogenesis and potential therapeutic implications for rare variants like AFX-like melanoma.
